# Assessing the impact of bacteriophages in the treatment of Salmonella in broiler chickens

**DOI:** 10.1080/20008686.2018.1539056

**Published:** 2018-10-30

**Authors:** Nehal M. Nabil, Maram M. Tawakol, Heba M. Hassan

**Affiliations:** Reference Laboratory for Veterinary Quality control on Poultry production, Animal health research institute, Giza, Egypt

**Keywords:** Bacteriophage, poultry population, Salmonella colonization

## Abstract

Salmonellosis is one of the main bacterial infections affecting commercial poultry, causing losses to poultry production, and posing a public health concern.

Samples from internal organs (liver, cecum and spleen) of one hundred diseased broiler chickens were collected and subjected to *Salmonella* isolation, identification and serotyping. *S. typhimurium* and *S. enteritidis* were selected from the isolated *Salmonella* to prepare bacteriophages from sewage water taken at broiler farms. An experimental infection of one day old specific pathogen free (SPF) chicks followed by treatment with the prepared bacteriophages isolated from both *Salmonella* was performed. Caecal samples from infected chicks were subjected at intervals to bacteriophage isolation and *Salmonella* quantitation. The effectiveness of bacteriophage treatments on *Salmonella* colonization in cecum of infected chicks increased after five successive doses. At 3 day post infection (dpi), cecal contents showed a marginal decrease in Salmonella loads with more reduction at 5 dpi. From 7 dpi to the end of the experiment at 15 dpi, all the chicks were cleared for both *Salmonella*.

The findings of this study demonstrate that bacteriophage treatment is efficacious in reducing *S. typhimurium* and *S. enteritidis* colonization in broiler chickens within a short period and could be used as an alternative to antibiotics.

## Introduction

Salmonellosis is one of the main infections affecting commercial poultry, causing losses to poultry production, and posing a public health concern [1]. Salmonella, the causative agent for salmonellosis, are Gram negative, rod shaped, facultative anaerobic bacteria causing gastroenteritis [2]. Fowl typhoid and pullorum disease, are widely distributed septicemic diseases, caused by *S. gallinarum* and *S. pullorum* respectively and infect primarily chickens and turkeys. These bacteria are transmitted mainly transovarially. Feces of infected birds, contaminated feed, water and litter can also be sources for infection. Clinical signs in chicks and poults include anorexia, dehydration, weakness, diarrhea and high mortality. Decreased egg production, fertility and hatchability are the most important clinical signs in mature birds. Gross and microscopic lesions include hepatitis, typhlitis, omphalitis, pneumonia, ophthalmitis salpingitis, synovitis and peritonitis [3].

Poultry gastrointestinal tract is considered as a major reservoir for various pathogenic bacteria that can cause cross-contamination of poultry meat and egg products [4]. For example, Salmonella can invade the intestinal epithelial cells and survive intracellularly within macrophages [5] and these intracellular Salmonella are not easily controlled by antibiotics. Bacteriophage control has received much attention as a potential treatment approach for bacterial infections [6] due to the emergence of antibiotic-resistant bacteria [7].

Bacteriophages are bacterial viruses [8], and as suggested by previous studies, possible alternatives to antibiotics for treatment of bacterial diseases [9]. Bacteriophages have no adverse effects on human or animal immune systems and does not affect the normal bacterial flora [10]. Phages are highly host specific, typically targetting a particular group of bacterial species [11]. Bacteriophages multiply inside the infected host cell in a so called lytic infection cycle, and are released from host cells by bacteriolysis. Bacteriophages infect bacteria by injecting their DNA across the bacterial envelope into the cytoplasm of the cell [12]. The injected phage DNA is replicated using the infected host cell metabolic machinery and the genes encoding the phage protein coat are expressed systematically [13].

The current study aims to isolate and identify *Salmonella* from internal organs of diseased broilers and assessing the effectiveness of crude bacteriophage preparations in the treatment of *Salmonella* colonization in the birds.

## Material and methods

### Sample collection and identification

A total of one hundred diseased broiler chickens (different ages) from 75 farms in Dakahlia Governorate (Egypt) were collected in the end of the 2017 and in the beginning of the year 2018. Samples from internal organs (liver, spleen and cecum) from each bird were collected aseptically, labeled and transported directly in an ice box to Reference Laboratory for Veterinary Quality control on Poultry production (RLQP) (Gamasa Lab) for Salmonella isolation.

Liver, cecum and spleen from each broiler chicken were pooled together as one sample. Salmonella was isolated and identified according to **ISO 6579 (2017)** [14] as follows: Samples were weighed and suspended in buffered peptone water (1:10 dilution) and incubated at 37ºC for 18 hours. The pre-enrichment broth after incubation was mixed and 0.1 ml of the broth was transferred into a tube containing 10 ml of Rappaport-Vassiliadis medium with soya (RVS broth). Another 1 ml of the pre-enrichment broth was transferred into a tube containing 10 ml of Muller-Kauffmann tetrathionate novobiocin broth (MKTTn broth). The inoculated RVS broth was incubated at 41.5°C for 24 hours and the inoculated MKTTn broth at 37°C for 24 hours. After incubation, a loop-full of material from the RVS broth and MKTTn was transferred and streaked separately onto the surface of Xylose Lysine Deoxycholate agar (XLD agar), Hektoen Enteric (HE agar), MacConkey’s agar and S-S agar respectively. The plates were incubated at 37°C for 24 hours then checked for growth of typical Salmonella colonies.

The isolates that were biochemically identified as Salmonella (methyl red, citrate utilization, triple sugar iron and catalase tests positive & negative to V-P, urease and indole tests) were subjected to serological identification according to Kauffman- white scheme [15] for determining somatic (O) and flagellar (H) antigens [16].

### Bacteriophages isolation and purification

*S. typhimurium* and *S. enteritidis* isolated from diseased broiler chickens in this study were selected and used for bacteriophage preparation. According to [10,13]; five sewage samples were collected from broiler farms and centrifuged at low speed (8496 g) for 10 minutes to remove solid particles and then filtered with 0.22 μm pore syringe filters. For bacteriophages enrichment, isolation and purification; *S. typhimurium* and *S. enteritidis* were grown overnight at 37°C in nutrient broth to obtain pure bacterial cultures. The next day, 0.1 ml of the overnight cultures were inoculated into 10 ml nutrient broth and incubated at 37°C for 3 hours with agitation to reach exponential phase. The sewage filter sample supernatant (4.5 ml) was mixed with 0.5 ml exponential phase bacterial cultures and 0.5 ml concentrated nutrient broth and was incubated at 37°C for 24 hours. After incubation samples were centrifuged at 8496 g for 10 minutes, supernatants were filtered with a 0.22 μm filter syringe and was used as enriched phage (EP) samples.

### Detection of bacteriophages: spot technique and plaque assay

The prepared bacteriophages were tested by spot testing to ensure the presence of lytic phages in the prepared enriched phage filtrates. One ml of cultured *S. typhimurium* and *S. enteritidis* was spread separately on nutrient agar plates, the excess fluid was removed and the plates were kept at room temperature to dry. 10 μl of the prepared enriched phage filtrate was spotted on the agar and allowed to dry. The agar plates were incubated at 37°C overnight for the detection of lytic spots (clearance zones) on to the agar plates according to Rahaman et al. [17].

Plaque assay was conducted according to Akhtar et al. [13], with some modifications; Ten-fold serial dilution was performed using 0.1 ml of the phage suspension (as prepared above). A single colony of overnight culture of *S. typhimurium* and *S. enteritidis* was inoculated separately into 5 ml of nutrient broth and incubated at 37°C for 3 hours. Semisolid agar (0.5%) was aliquoted (3 ml) into tubes placed at 45°C in a water bath, 0.1 ml phage and 0.5 ml *S. typhimurium* and *S. enteritidis* separately were added to semisolid agar tubes, then mixed and poured onto nutrient agar plates. The plates were allowed to cool and incubated overnight at 37°C. Plaques were counted and the number of phages was determined as plaque forming unit (per ml?) (PFU).

### In vivo assay of bacteriophage treatment in experimentally infected chicks

Ethical approval: The animal experiment was performed according to the legally approved protocol [AHRI (29) 23/11/2017] of the Animal Health and Research Institute (AHRI), Giza, Egypt. Isolation experiments were performed in separated cages at a biosecurity level- two (BSL-2) animal facility.

Experimental design: One hundred and twenty SPF chicks (one day old) were housed in air-filtered isolation cabinets in Reference Laboratory for Veterinary Quality Control on Poultry production (RLQP). From Table 1, the chicks under experiment (120 chicks) were divided into four groups, each group contained 30 chicks. Each group of birds was placed individually, provided feed and water ad libitum. The chicks were maintained at an age-appropriate temperature until the end of the experiment.

**Table 1.** experimental design of bacteriophage treatments in (SPF) chicks challenged with *S*. typhimurium and *S*. enteritidis.

		Doses				
Group	NO. of chicks	Bacteriophage	Salmonella	Treatment schedule of bacteriophage (days)	Age of Salmonella infection	Time of euthanasia (days)
Group (1)^a^	30	–	–	–	–	5, 7, 9, 11, 14, 17
Group (2)^b^	30	0.1ml 1.18 × 10^11^PFU/chick	0.1ml 10^5^ CFU/chick (S.T.)	1, 2, 3, 6, 8, 10, 13, 15	2nd day	5, 7, 9, 11, 14, 17
Group (3)^c^	30	0.1 ml 1.03 × 10^12^PFU/chick	0.1 ml 10^5^ CFU/chick (S.E.)	1, 2, 3, 6, 8, 10, 13, 15	2nd day	5, 7, 9, 11, 14, 17
Group (4)^d^	(A) 15	–	0.1ml 10^5^ CFU/chick (S.T.)	–	2nd day	5, 7, 9, 11, 14, 17
	(B) 15	–	0.1ml 10^5^ CFU/chick (S.E.)	–	2nd day	5, 7, 9, 11, 14, 17

^a^ group (1) control negative

^b^ group (2) bacteriophage treated and infected with *S*. Typhimurium

^c^ group (3) bacteriophage treated and infected with *S*. Enteritidis

^d^ group (4) control positive infected with *S*. Typhimurium (part A) and *S*. Enteritidis (part B).

Bacteriophages of *S. typhimurium* that were prepared in this study were administered orally on several occasions; in all chicks of group 2 at days 1, 2, 3, 6, 8, 10, 13 and 15 with a 0.1 ml solution containing 1.18 × 10^11^ PFU phages/chick in 0.1 ml. Subsequently, all chicks in this group were challanged orally at day two with a single dose of 10^5^ CFUs *S. typhimurium* in a total volume of 0.1 ml. Group 3 followed a similar regime, except that the orally administered bacteriophages (1.03 × 10^12^ PFU/chick) were amplified and isolated from *S. enteritidis* cultures and the chicks were challenged orally at day two with a single dose of 10^5^ CFUs *S. enteritidis*.

Group 4 was a positive control for both *Salmonella*. Fifteen chicks were inoculated orally at day two with a single dose of 0.1 ml culture of *S. typhimurium* at a concentration of 10^5^ CFU/chick. The remaining fifteen chicks were kept in another separate isolator and inoculated orally with a single dose of 0.1 ml culture of *S. enteritidis* at a concentration of 10^5^ CFU/chick. Group 1 was left as a negative control and not inoculated with bacteriophages or *Salmonella*.

Five chicks from group 1, 2, 3 and 4 respectively, were euthanized at days 5, 7, 9, 11, 14 and 17. The experiment was terminated on day 17.

### Isolation of Salmonella and bacteriophages from chicks during experiment

Liver, spleen and cecum of the necropsied chicks were subjected to Salmonella isolation and identification according to **ISO 6579 (2017)** [17]; the representative colonies of *S. typhimurium* and *S. enteritidis* were confirmed by slide agglutination tests with poly (O), poly (H), and serotype-specific antisera. The Bacteriophages were isolated from caecal contents of group 2 and 3; a suspension of cecal contents (1 gram caecal content: 9 ml physiological saline, pH 7.4) was centrifuged at (10,000 rpm) for 10 minutes to remove any debris. The supernatant was then filtered with 0.22 μm filter syringe to remove any remaining bacteria. Finally 10 μl of the filtrate was used for spot testing to detect the presence of bacteriophages according to Rahaman et al. [17].

### Quantitative detection of Salmonella in caecal contents of chicks

Total DNA was extracted from caecal contents of all chicks in groups 2 & 3 and one chick from group 1 and 4 at each time point using QIAamp DNA Mini kit (Qiagen, Germany, GmbH) with modifications from the manufacturer’s recommendations (the rest of chicks in group 1 and 4 were examined by plating on XLD agar [18]). Briefly, 200 µl of the caecal content suspension was incubated with 10 µl of proteinase K and 200 µl of lysis buffer at 56°C for 10 min. After incubation, 200 µl of 100% ethanol was added to the lysate. The sample was then washed and centrifuged following the manufacturer’s recommendations. DNA was eluted with 100 µl of elution buffer provided in the kit. Oligonucleotide primers and probes that used were provided from Metabion (Germany) listed in Table 2. DNA amplifications were performed in a final volume of 25 µl containing 3 µl of DNA template, 12.5 µl of 2 × QuantiTect Probe RT-PCR Master Mix, 8.875 µl PCR grade water, 0.25 µl of each primer (50 pmol conc.) and 0.125 µl of each probe (30 pmol conc.). Primary denaturation was performed at 94°C for 15 min, followed by 40 cycles of denaturation at 94°C for 15 s, annealing at 49°C for 30 s and extension at 72°C for 10 s. The bacterial concentration of samples was determined with cfu/g. A known standard (of Known CFU/g) was ten-fold serially diluted, then DNA was extracted separately from the last 5 dilutions and tested by rt-PCR with the same conditions of the unknown samples. The standard was included in all PCR runs. The reaction was done in Stratagene MX3005P real time PCR machine (that can show the standard quantity with 3 decimals).

**Table 2.** Oligonucleotide primers and probes used in this study.

Gene	Primer/probe sequence 5ʹ–3’	Reference
*inv*A	GCGTTCTGAACCTTTGGTAATAA	Daum et al. (19)
	CGTTCGGGCAATTCGTTA	
	5′-FAM-TGGCGGTGGGTTTTGTTGTCTTCT-TAMRA-3′	

## Results

### Incidence of Salmonella isolation

Eight Salmonella clones were isolated from 100 diseased broiler chickens in Dakahlia Governorate with an incidence of 8%. The serotyping of the isolated *Salmonella* revealed (2) *S. typhimurium*, (2) *S. kentucky*, (1) *S. enteritidis*, (1) *S. molade*, (1) *S. inganda* and (1) *S. apyeme*.

### Bacteriophage isolation, detection and enumeration

**Figure 1.**
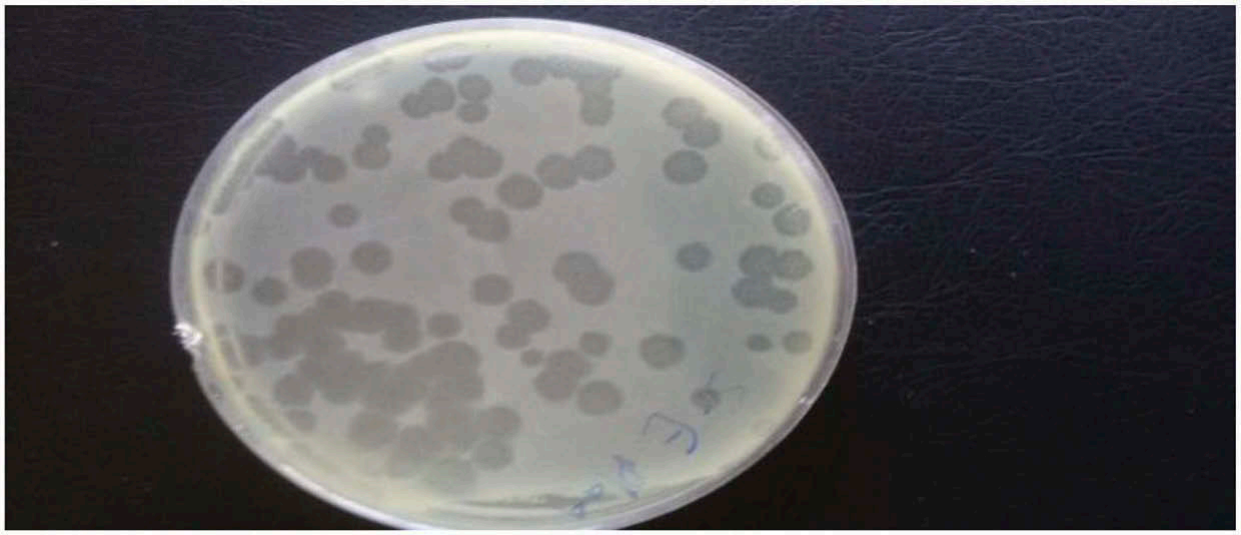
Plaque assay for bacteriophage enumeration.

Bacteriophages of *S. typhimurium* and *S. enteritidis* were prepared from environmental sewage samples from broiler farms. Spot testing confirmed the presence of specific lytic bacteriophages through the appearance of clearance zones (lytic spots) in plates coated with respective *Salmonella* species. Bacteriophages were also enumerated using plaque assay for both *Salmonella* species to prepare the phage samples needed for the experiment (Figure 1) and the results revealed that the concentration of bacteriophages infecting *S. typhimurium* was 118 × 10^10^ PFU/ml and 103 × 10^11^ PFU/ml for *S. enteritidis*.

### Clinical signs and bacteriophage isolation

**Figure 2.**
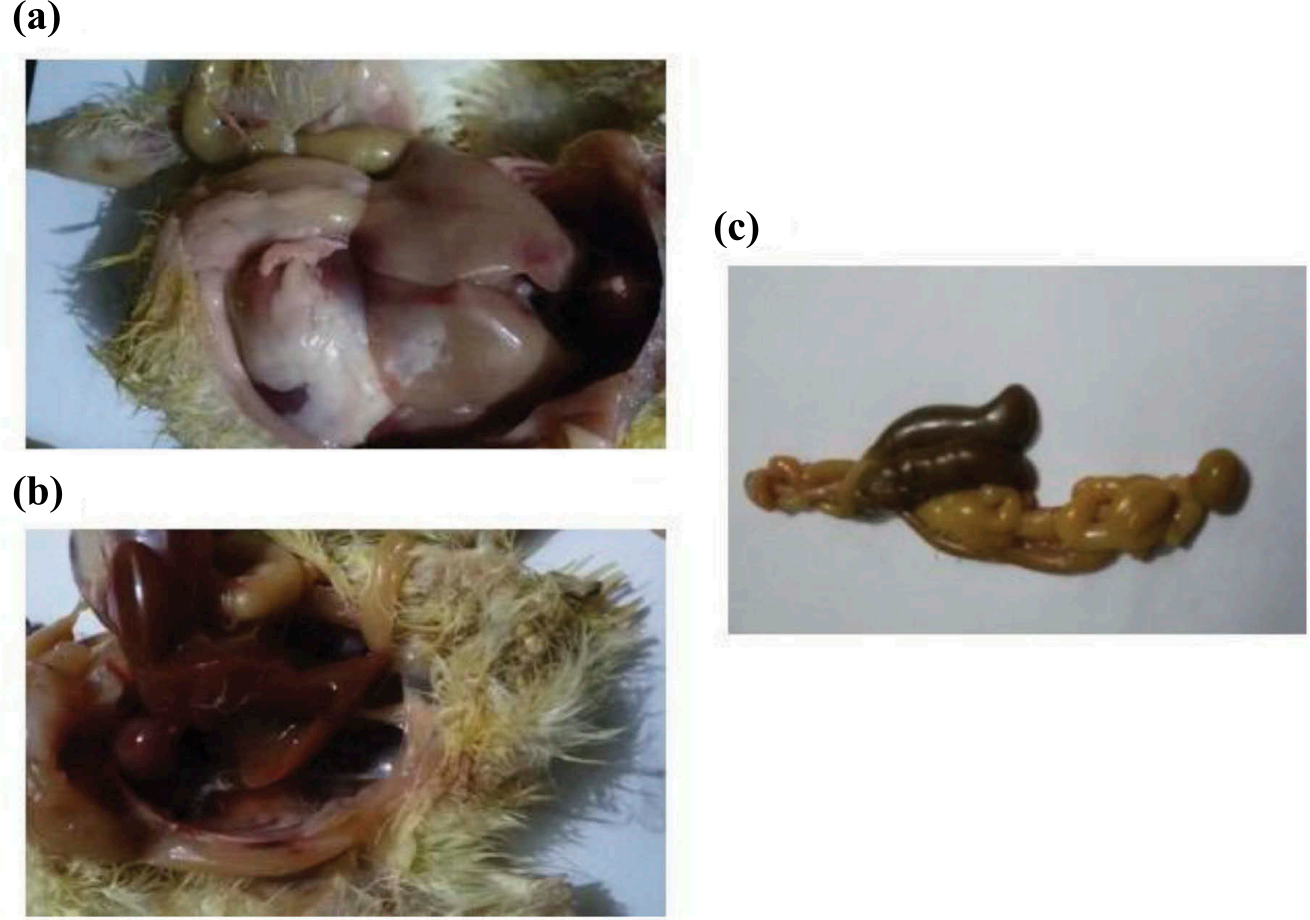
(a) hemorrhagic patches in liver with distention of intestine with diarrhea (b) congestion of internal organs with pasting vent (c) enlargement and distention of the two ceci with diarrhea.

All chicks in group 1 (negative control) showed no clinical signs and no post mortem findings. The chicks in group 4 part A and part B (positive controls for *S. typhimurium* and *S. enteritidis* infections respectively) showed depression, loss of appetite, inability to stand, diarrhea and pasting vent. Congestion of internal organs, hemorrhages in liver, unabsorbed yolk sac in some chicks and enlargement of two caeci with diarrhea were observed (Figure 2). The mortalities appeared after 3 days of *Salmonella* and the incidence of mortalities at the end of the experiment were 30% for *S. enteritidis* and 20% for *S. typhimurium*.

In the infected groups 2 and 3 that were treated with bacteriophages at different time points (days 1, 2, 3, 6, 8, 10, 13 and 15) showed depression and diarrhea after 2 days of infection. These signs disappeared gradually and the postmortem findings at 3 dpi were enlargement of two ceci with diarrhea, congestion in liver and few chicks showed hemorrhages in heart. At 7 dpi until the end of experiment, the activity of the chicks increased and the diarrhea stopped.

Bacteriophages were isolated from caecal contents of sacrificed chicks in group 2 and 3; spot tests confirmed the presence of *Salmonella* specific bacteriophages, producing distinct clearance zones on plates with the two different *Salmonella* species respectively.

### Salmonella isolation, identification and quantitative detection

All chicks in group 1 (uninfected negative control) were negative for *Salmonella* colonization as determined by plating on XLD agar. All chicks in group 4-A and 7 chicks in group 2 till the end of the experiment were positive for *S. typhimurium* and the species was confirmed using serotype-specific antisera.

Similarily, all chicks in group 4-B and 7 chicks in group 3 were positive for *S. enteritidis* and again, the species was confirmed using serotype-specific antisera. All chicks in group 4 (infection control) were tested positive for *Salmonella* by plating on XLD agar.

Quantitative Real time PCR (RT- PCR) was used as a rapid and accurate technique to determine *Salmonella* loads in caecum of necropsied chicks in groups 2 and 3 and to investigate the significance and the effectiveness of bacteriophage treatments on *Salmonella* colonization. Tables 3 & 4 show that bacteriophage treatments start to reduce *S. typhimurium* (group 2) and *S. enteritidis* (group 3) colonization in cecum after four successive doses. At the 3 dpi of age, cecal contents of all sacrificed chicks showed a slight decrease in *Salmonella* colonization in comparison to positive control groups. In the 5 dpi colonization of both *Salmonella* reduced in comparison with the 3 dpi. From the beginning of the 7 dpi till the end of the experiment at 15 dpi(after five successive doses of bacteriophage treatments) all the chicks showed no colonization for both *Salmonella* in caecum which suggest that the bacteriophage treatment is successful in treatment of *Salmonella*..

**Table 3.** Quantitative detection of *S*. typhimurium colonization in cecum of chicks under experiment.

Sample code	Group No.	Age of chicks	Results	Ct.	Conc. (CFU/gm)
T1	2	5 days	Positive	24.35	1.413 × 10^2^
T2	2	5 days	Positive	23.38	2.778 × 10^2^
T3	2	5 days	Positive	25.78	5.213 × 10^2^
T4	2	5 days	Positive	22.95	3.749 × 10^2^
T5	2	5 days	Positive	23.10	3.376 × 10^2^
**T6 (negative control)**	**1**	**5 days**	**Negative**	**No CT**	**–**
**T8 (positive control)**	**4 A**	**5 days**	**Positive**	**22.36**	**5.656 × 10^2^**
T9	2	7 days	Positive	23.62	2.350 × 10^2^
T10	2	7 days	Negative	No CT	–
T11	2	7 days	Negative	No CT	–
T12	2	7 days	Positive	22.85	4.019 × 10^2^
T13	2	7 days	Negative	No CT	–
**T14(negative control)**	**1**	**7 days**	**Negative**	**No CT**	**–**
**T16(positive control)**	**4A**	**7 days**	**Positive**	**21.70**	**8.960 × 10^2^**
T17	2	9 days	Negative	No CT	–
T18	2	9 days	Negative	No CT	–
T19	2	9 days	Negative	No CT	–
T20	2	9 days	Negative	No CT	–
T21	2	9 days	Negative	No CT	–
**T22(negative control)**	**1**	**9 days**	**Negative**	**No CT**	–
**T24(positive control)**	**4A**	**9 days**	**Positive**	**20.45**	**2.142 × 10^3^**
T25	2	11 days	Negative	No CT	–
T26	2	11 days	Negative	No CT	–
T27	2	11 days	Negative	No CT	–
T28	2	11 days	Negative	No CT	–
T29	2	11 days	Negative	No CT	–
**T30(negative control)**	**1**	**11 days**	**Negative**	**No CT**	–
**T32(positive control)**	**4A**	**11 days**	**Positive**	**20.63**	**1.889 × 10^3^**
T33	2	14 days	Negative	No CT	–
T34	2	14 days	Negative	No CT	–
T35	2	14 days	Negative	No CT	–
T36	2	14 days	Negative	No CT	–
T37	2	14 days	Negative	No CT	–
**T38(negative control)**	**1**	**14 days**	**Negative**	**No CT**	–
**T40(positive control)**	**4A**	**14 days**	**Positive**	**19.84**	**3.277 × 10^3^**
T41	2	17 days	Negative	No CT	–
T42	2	17 days	Negative	No CT	–
T43	2	17 days	Negative	No CT	–
T44	2	17 days	Negative	No CT	–
T45	2	17 days	Negative	No CT	–
**T46(negative control)**	**1**	**17 days**	**Negative**	**No CT**	–
**T48(positive control)**	**4A**	**17 days**	**Positive**	**19.20**	**5.119 × 10^3^**

T: Typhimurium Ct: cycle threshold conc.: concentration.

**Table 4.** Quantitative detection of *S*. enteritidis colonization in cecum of chicks under experiment.

Sample code	Group No.	Age of chicks	Results	Ct.	Conc. (CFU/gm)
E1	3	5 days	Positive	22.49	7.415 × 10^3^
E2	3	5 days	Positive	23.81	2.999 × 10^3^
E3	3	5 days	Positive	24.20	2.296 × 10^3^
E4	3	5 days	Positive	24.57	1.781 × 10^3^
E5	3	5 days	Positive	22.01	1.030 × 10^4^
**E6 (negative control)**	**1**	**5 days**	**Negative**	**No CT**	**–**
**E8 (positive control)**	**4B**	**5 days**	**Positive**	**22.86**	**5.753 × 10^3^**
E9	3	7 days	Positive	21.45	1.513 × 10^4^
E10	3	7 days	Negative	No CT	–
E11	3	7 days	Positive	20.86	2.267 × 10^4^
E12	3	7 days	Negative	No CT	–
E13	3	7 days	Negative	No CT	–
**E14(negative control)**	**1**	**7 days**	**Negative**	**No CT**	**–**
**E16 (positive control)**	**4B**	**7 days**	**Positive**	**22.05**	**1.003 × 10^4^**
E17	3	9 days	Negative	No CT	–
E18	3	9 days	Negative	No CT	–
E19	3	9 days	Negative	No CT	–
E20	3	9 days	Negative	No CT	–
E21	3	9 days	Negative	No CT	–
**E22(negative control)**	**1**	**9 days**	**Negative**	**No CT**	-
**E24(positive control)**	**4B**	**9 days**	**Positive**	**21.32**	**1.654 × 10^4^**
E25	3	11 days	Negative	No CT	–
E26	3	11 days	Negative	No CT	–
E27	3	11 days	Negative	No CT	–
E28	3	11 days	Negative	No CT	–
E29	3	11 days	Negative	No CT	–
**E30(negative control)**	**1**	**11 days**	**Negative**	**No CT**	**–**
**E32(positive control)**	**4B**	**11 days**	**Positive**	**20.74**	**2.461 × 10^4^**
E33	3	14 days	Negative	No CT	–
E34	3	14 days	Negative	No CT	–
E35	3	14 days	Negative	No CT	–
E36	3	14 days	Negative	No CT	–
E37	3	14 days	Negative	No CT	–
**E38(negative control)**	**1**	**14 days**	**Negative**	**No CT**	**–**
**E40(positive control)**	**4B**	**14 days**	**Positive**	**20.15**	**3.689 × 10^4^**
E41	3	17 days	Negative	No CT	–
E42	3	17 days	Negative	No CT	–
E43	3	17 days	Negative	No CT	–
E44	3	17 days	Negative	No CT	–
E45	3	17 days	Negative	No CT	–
**E46(negative control)**	**1**	**17 days**	**Negative**	**No CT**	**–**
**E48(positive control)**	**4B**	**17 days**	**Positive**	**18.21**	**1.395 × 10^5^**

E: Enteritidis Ct: cycle threshold conc.: concentration.

## Discussion

In the current study the incidence of *Salmonella* was similar to that previously reported by Abd El-Ghany et al. [20], who isolated *Salmonella* from diseased broilers in Kalubia Governorate- Egypt with an incidence of (7.03%). Serotyping in that study revealed the presence of *S. typhimurium, S. kentucky* and *S. enteritidis*, species that were also present in the current study. Also, some authors such as Jafari et al. [21], and Boonmara et al. [22], previously reported similar incidence levels of Salmonella, which were (5.8%) and (6.65%) respectively. In a previous study performed by Mohamed et al. [23], in Kafr Elsheik Governorate- Egypt, Salmonella was isolated from broiler chickens with an incidence of (2.5%) and this percentage was lower than the current study. A higher percentage of Salmonella isolation was recorded and isolated with an incidence of (12%).

Bacteriophages in this study were isolated and prepared from sewage of poultry farms. Presence of *Salmonella* specific bacteriophages has previously been reported from excretion sewage of commercial broiler houses [24]. Meanwhile Andreatti Filho et al. [18], isolated bacteriopgages from environmental drag swabs in commercial broiler houses. Spot tests were used in this study to confirm the presence of specific bacteriophages in the preparations, other studies such as Rahaman et al. [17], have used similar strategies to test for the presence of specific bacteriophages. Further, the presence of bacteriophages during the experiment was verified by isolation of phages from caecal contents of chicks treated with bacteriophages, a similar method used in previous studies [25,26].

In this study bacteriophages were administrated to chicks before oral *Salmonella* infections followed by 4 successive phage treatments after the bacterial challenge. Although *Salmonella* were still able to colonize the chicks, bacterial loads decreased after four successive phage treatments. After the 5th dose no bacteria were detected indicating that the chicks treated with phages were cured of *Salmonella*. This effect is most likely due to the lytic effect of the administered bacteriophages against *Salmonella*. Moreover, bacteriophages have previously been used to control intracellular pathogens [27]. Some bacteriophages can be efficacious in reducing *S. enteritidis* colonization in poultry during a short period [18]. In our study, we were able to clear *Salmonella* from infected chicks after successive phage treatments applied within a short time period after infection. A previous study reported similar results as the present study, where bacteriophages was reported as a factor resulting in reduced *Salmonella* CFU/g caecal content and reduced *Salmonella* colonization in broiler chicks after 5 days of treatment [26]. Bacteriophages have also been reported to reduce the viability of *S. typhimurium* in chicken cecum for up to 12 hours after inoculation [25].

Thus, bacteriophage treatment has a significant effect in reducing colonization of both *S. enteritidis* and *S. typhimurium* in cecum of broiler chickens as previously suggested by Atterbury et al. [28].

In conclusion, the increasing resistance of *Salmonella* strains to the most used antibiotics in broiler farms is considered an important problem leading to high economic losses to the poultry industry. Antibiotic treatments does not only kill pathogenic bacteria but also affect the normal micro flora, potentially leading to secondary infections. Hence, novel bacteriophage treatments, as shown in this study, show great promise for the treatment of bacterial infections in the poultry industry. Phage therapy has reduced side effects compared to traditional antibiotic treatments due to the specificity of phages. Our results suggest that bacteriophage treatment is efficacious in reducing *S. typhimurium* and *S. enteritidis* colonization in cecum of broiler chicks within a short period after oral administration of the prepared bacteriophage. Treatments with five successive doses was highly effective and cleared the *Salmonella* infection in new born chicks, therefore it could be recommended to administer bacteriophages orally with five consecutive doses to reduce the *Salmonella* load in poultry farms. Further studies with a wider scope is recommended to investigate the implementation of bacteriophage administration programs in poultry farms as a routine treatment, phage typing and the specificity of the prepared phage(s) of *S. typimurium* will be tested on *S. enteritidis* and vice versa.
